# Comprehensive analysis of oncogenic fusions in mismatch repair deficient colorectal carcinomas by sequential DNA and RNA next generation sequencing

**DOI:** 10.1186/s12967-021-03108-6

**Published:** 2021-10-17

**Authors:** Jing Wang, Ruiyu Li, Junjie Li, Yuting Yi, Xiaoding Liu, Jingci Chen, Hui Zhang, Junliang Lu, Cami Li, Huanwen Wu, Zhiyong Liang

**Affiliations:** 1 Department of Pathology, Peking Union Medical College Hospital, and Molecular Pathology Research Center, Chinese Academy of Medical Sciences and Peking Union Medical College, Beijing, 100730 China; 2 Geneplus-Beijing Institute, Beijing, China

**Keywords:** Mismatch repair, Colorectal carcinoma, RNA next generation sequencing, Gene fusion

## Abstract

**Background:**

Colorectal carcinoma (CRC) harboring oncogenic fusions has been reported to be highly enriched in mismatch repair deficient (dMMR) tumors with *MLH1* hypermethylation (*MLH1*^me+^) and wild-type *BRAF* and *RAS*. In this study, dMMR CRCs were screened for oncogene fusions using sequential DNA and RNA next generation sequencing (NGS).

**Results:**

Comprehensive analysis of fusion variants, genetic profiles and clinicopathological features in fusion-positive dMMR CRCs was performed. Among 193 consecutive dMMR CRCs, 39 cases were identified as *MLH1*^me+^ *BRAF/RAS* wild-type. Eighteen fusion-positive cases were detected by DNA NGS, all of which were *MLH1*^me+^ and *BRAF/RAS* wild-type. RNA NGS was sequentially conducted in the remaining 21 *MLH1*^me+^ *BRAF/RAS* wild-type cases lacking oncogenic fusions by DNA NGS, and revealed four additional fusions, increasing the proportion of fusion-positive tumors from 46% (18/39) to 56% (22/39) in *MLH1*^me+^ *BRAF/RAS* wild-type dMMR cases. All 22 fusions were found to involve RTK-RAS pathway. Most fusions affected targetable receptor tyrosine kinases, including *NTRK1*(9/22, 41%), *NTRK3*(5/22, 23%), *ALK*(3/22, 14%), *RET*(2/22, 9%) and *MET*(1/22, 5%), whilst only two fusions affected mitogen-activated protein kinase cascade components *BRAF* and *MAPK1*, respectively. *RNF43* was identified as the most frequently mutated genes, followed by *APC*, *TGFBR2*, *ATM*, *BRCA2* and *FBXW7*. The vast majority (19/22, 86%) displayed alterations in key WNT pathway components, whereas none harbored additional mutations in RTK-RAS pathway. In addition, fusion-positive tumors were typically diagnosed in elder patients and predominantly right-sided, and showed a significantly higher preponderance of hepatic flexure localization (*P* < 0.001) and poor differentiation (*P* = 0.019), compared to fusion-negative *MLH1*^me+^ CRCs.

**Conclusions:**

We proved that sequential DNA and RNA NGS was highly effective for fusion detection in dMMR CRCs, and proposed an optimized practical fusion screening strategy. We further revealed that dMMR CRCs harboring oncogenic fusion was a genetically and clinicopathologically distinctive subgroup, and justified more precise molecular subtyping for personalized therapy.

**Supplementary Information:**

The online version contains supplementary material available at 10.1186/s12967-021-03108-6.

## Background

Colorectal carcinoma (CRC) represents one of the most common malignancies worldwide, ranking third and fifth for cancer-related deaths in United States and China, respectively [1]. Nowadays, there is an increasing recognition that AJCC-TNM staging is insufficient for personalized therapy. The molecular heterogeneity of CRCs has been widely emphasized, and proved to be of critical prognostic and therapeutic significance.

Oncogenic fusions have long been well-recognized as not only diagnostic or prognostic markers, but also potential therapeutic targets in different cancer types, including CRCs [2]. With the emerging introduction of fusion targeted therapy, efficient and accurate detection of druggable gene fusions is becoming increasingly important for clinical decision making. Fusion gene diagnosis was traditionally performed by fluorescence in situ hybridization (FISH) or quantitative real-time polymerase chain reaction (RT-PCR) assay. Despite the high sensitivity, these methods typically test for only one specific fusion gene, and provide very limited information of the fusion partners and breakpoints [3]. Targeted DNA-based next generation sequencing (NGS) has been proved to effectively detect common oncogenic fusions with high confidence. However, some gene fusions of high clinical relevance may be missed due to the insufficient coverage of large introns and blind-spot within the targeted areas [4]. By comparison, RNA NGS can overcome many of these limitations by conducting genome-wide inspection of gene fusions with nucleotide-level resolution of genomic breakpoints, identifying both known and novel fusion genes, and delineating the fusion transcripts directly at the mRNA level [3, 5]. Currently, RNA NGS has been proved to be an indispensable testing in routine diagnostics for sarcoma[6], and an important complement to DNA NGS for high yield detection of targetable gene fusions in non-small cell lung cancers [7, 8]. Nevertheless, reports regarding RNA NGS in fusion gene diagnosis of other cancers, including CRCs, are still limited.

Previously, oncogenic fusions were considered to be rare molecular events in CRCs, presenting in less than 1% of unselected patients [9]. Due to the extremely low prevalence, universal assessment for gene fusions utilizing high-throughput methods in routine clinical practice could be expensive and time-consuming. A practical and efficient strategy to screen for such rare but clinically critical molecular alteration was highly warranted. Notably, we and others have recently uncovered that gene fusions were nearly exclusively detected, and significantly enriched in a specific molecular subtype of mismatch repair deficient (dMMR) CRCs, characterized by hypermethylated *MLH1* (*MLH1*^me+^) and wild-type *BRAF/RAS* [9–11]. A preliminary screening protocol using routine molecular pathological assays has also been proposed by us [10]. In the present study, we enlarged the sample size and incorporated RNA NGS in complement to DNA NGS for fusion detection, aiming to improve our prior fusion screening strategy, and achieve more comprehensive understanding of this rare CRC subtype.

In this study, DNA NGS was performed in a retrospective consecutive cohort of dMMR CRCs, whilst RNA NGS was sequentially conducted in *MLH1*^me+^ *BRAF/RAS* wild-type dMMR CRCs lacking oncogenic fusions by DNA NGS. We revealed that additional RNA NGS could efficiently enhance fusion detection, and accordingly proposed an optimizing strategy to screen for potential targetable gene fusions in CRCs using combined DNA NGS and RNA NGS. A complete review of fusion genes and variants was presented. Molecular genetic features and clinicopathological features in dMMR CRC with oncogenic fusions were also analyzed.

## Materials and methods

### Patient selection

This retrospective study involved consecutive CRC cases (n = 2230) from July 2015 until June 2020 in Peking Union Medical College Hospital (PUMCH). All patients with materials included in the study underwent a partial colectomy for primary CRC. None of the patients were known to have received neoadjuvant therapy or tyrosine kinase inhibitor therapy prior to surgery. This study was approved upon ceding review by the PUMCH Institutional Review Board for review.

### DNA and RNA extraction

DNA and RNA were isolated from formalin-fixed paraffin-embedded (FFPE) CRC specimens using Direct FFPE DNA Kit (Qiagen #A31133) and RNeasy FFPE Kit (Qiagen #73504), respectively, according to the manufacturer’s protocols.

### DNA NGS and determination of mutational significance

DNA targeted sequencing was performed using hybrid capture-based targeted next-generation sequencing (NGS) as previously described. Barcoded libraries were hybridized to our customized panel of 1,021 genes containing whole exons, selected introns of 288 genes and selected regions of 733 genes (Additional file 1: Table S1). The libraries were prepared and sequenced to a uniform median depth (> 500×). Genomic alterations, including single nucleotide variants, small insertions and deletions, copy number alterations, and gene fusions/rearrangements, were compared against each patient’s corresponding normal sample. After removing raw reads containing adaptor sequences, those with more than 50% low-quality base reads, or those with more than 50% N bases, together with their mate pair, reads were mapped to the reference human genome (hg19) using the Burrows-Wheel Aligner (http://bio-bwa.sourceforge.net/) with default parameters. Duplicate reads were identified and marked with Picard’s Mark Duplicates tool (https://software.broadinstitute.org/gatk/documentation/tooldocs/4.0.3.0/picard_sam_markduplicates_MarkDuplicates.php) for tumor and germline DNA data and were clustered according to UID and position of the template fragments for cfDNA data. Errors introduced by PCR or sequencing were corrected according to clustered reads. Local realignment and base quality recalibration were performed using The Gene Analysis Toolkit (https://www.broadinstitute.org/gatk/). Somatic single-nucleotide variations (SNVs) were called using the MuTect2 algorithm (https://software.broadinstitute.org/gatk/documentation/tooldocs/3.8-0/org_broadinstitute_gatk_tools_walkers_cancer_m2_MuTect2.php). Candidate mutations were filtered if: (1) more than 10 reads with insertions/deletions in an 11-bp window were centered; (2) the matched germline DNA control sample carried ≥ 3% or ≥ 2% alternate allele reads, and the sum of quality scores was above 80; (3) the candidate was found in dbsnp (version 138, https://www.ncbi.nlm.nih.gov/SNP/) but not listed in the COSMIC database; (4) the candidate was supported by fewer than five high-quality reads (base quality ≥ 30, mapping quality ≥ 30); or (5) the allele frequency was less than 1%. Insertions or deletions of small fragments (indels) were called using MuTect2 with default parameters. Variants detected in matched control samples with three or more reads indicating indels at the same location or in the 40-bp flanking regions of experimental samples or residing near regions with low complexity or short tandem repeats were removed. Remaining mutations were considered validated somatic variants. CNVs in tumor DNA was called using The Contra algorithm (http://contra-cnv.sourceforge.net). Genomic DNA sequencing libraries were prepared using the protocols recommended The KAPA Library Preparation Kit (Kapa Biosystems, Wilmington, MA, USA). Genomic DNA sequencing libraries were prepared using the protocols recommended The KAPA Library Preparation Kit (Kapa Biosystems, Wilmington, MA, USA). The libraries were hybridized to custom-designed probes covering 1021 genes (Integrated DNA Technology, Coralville, IA, USA), including selected for the detection of genomic rearrangements. Genomic rearrangements were identified by the software developed in-house analyzing chimeric read pairs. MSI status was determined using MSIsensor (v0.2), which reported the percentage of unstable somatic microsatellites through a Chi-square test on predefined microsatellite regions covered by our panel. The average sequencing depth for the target regions of the tumor samples was 2447×, and 99.0% of the average coverage of the targeted regions was more than 200×, which were qualified for variant calling and the MSI analysis.

Mutations of oncogenes were filtered according to the corresponding documentation in the Catalog of Somatic Mutations in Cancer [12] and OncoKB [13] annotation. Mutational significance of tumor suppressor genes was determined according to protocols described in our previous study [14], and only “predicted deleterious” mutations were included in the analysis.

### RNA NGS

NEBNext rRNA Depletion Kit (Human/Mouse/Rat) (NEB #Z1955E) was chosen to remove the targeted ribosomal RNA (rRNA). All RNA with a percentage of RNA fragments > 200 nucleotides (DV200) ≤ 50% skipped fragmentation and proceeded to library preparation. After rRNA depletion and fragmentation, cDNA synthesis and NGS library preparation were performed using NEBNext^®^ Ultra™ II Directional RNA Library Prep Kit (NEB#E7760L). The library was quantitated using Qubit 3.0 (life Invitrogen, USA) and quality was assessed with LabChip GX Touch (PerkinElmer, USA). After removal of terminal adaptor sequences and low-quality data by using fastp (version: 0.19.5) [15] and removal rRNA reads through aligning clean reads to rRNA database (download from NCBI) by using bowtie2 (version:2.2.8) [16], clean reads without known rRNA were aligned to the reference human genome (hg19) through STAR (version 020201) [17]. Fusions were detected by a customized version of Arriba 1.1.0. and annotated by in house software annoFilterArriba (version:1.0.0) with NCBI release 104 database. All final candidate fusions were manually verified with the integrative genomics viewer browser. A series of quality control metrics was computed by using RNA-SeQC assessment [18]. A threshold of ≥ 80 million mapped reads and ≥ 10 million junction reads per sample was set.

### *MLH1* promoter hypermethylation analysis

*MLH1* promoter hypermethylation analysis was performed using methylation-specific PCR, with the protocol as previously described [10, 14].

### Statistical methods

Continuous variables were presented as mean ± standard deviation, and categorical variables were expressed as percentages. Chi-square test, Fisher’s exact test, or Mann–Whitney test was used when appropriate for comparison between dMMR CRCs with fusion and dMMR CRCs without fusion. Statistical processing was performed using SPSS version 24 (SPSS Inc., Chicago, IL, USA) and *P* < 0.05 (two-sided) was considered statistically significant.

## Results

### Screening for *MLH1*-hypermethylated dMMR CRC cases

Of the 2230 cases in the consecutive CRC cohort, 193 (9%) cases showed absent immunohistochemical (IHC) staining in any of four MMR proteins (MLH1, MSH2, MSH6 and PMS2), and were identified as dMMR tumors. One hundred and forty-three cases showing lost MLH1/PMS2 expression were subjected to methylation-specific PCR. Of these, ninety-one cases (91/143, 64%) presented *MLH1* promoter hypermethylation.

### Complete review of gene fusions detected by sequential DNA and RNA NGS

**Fig. 1:**
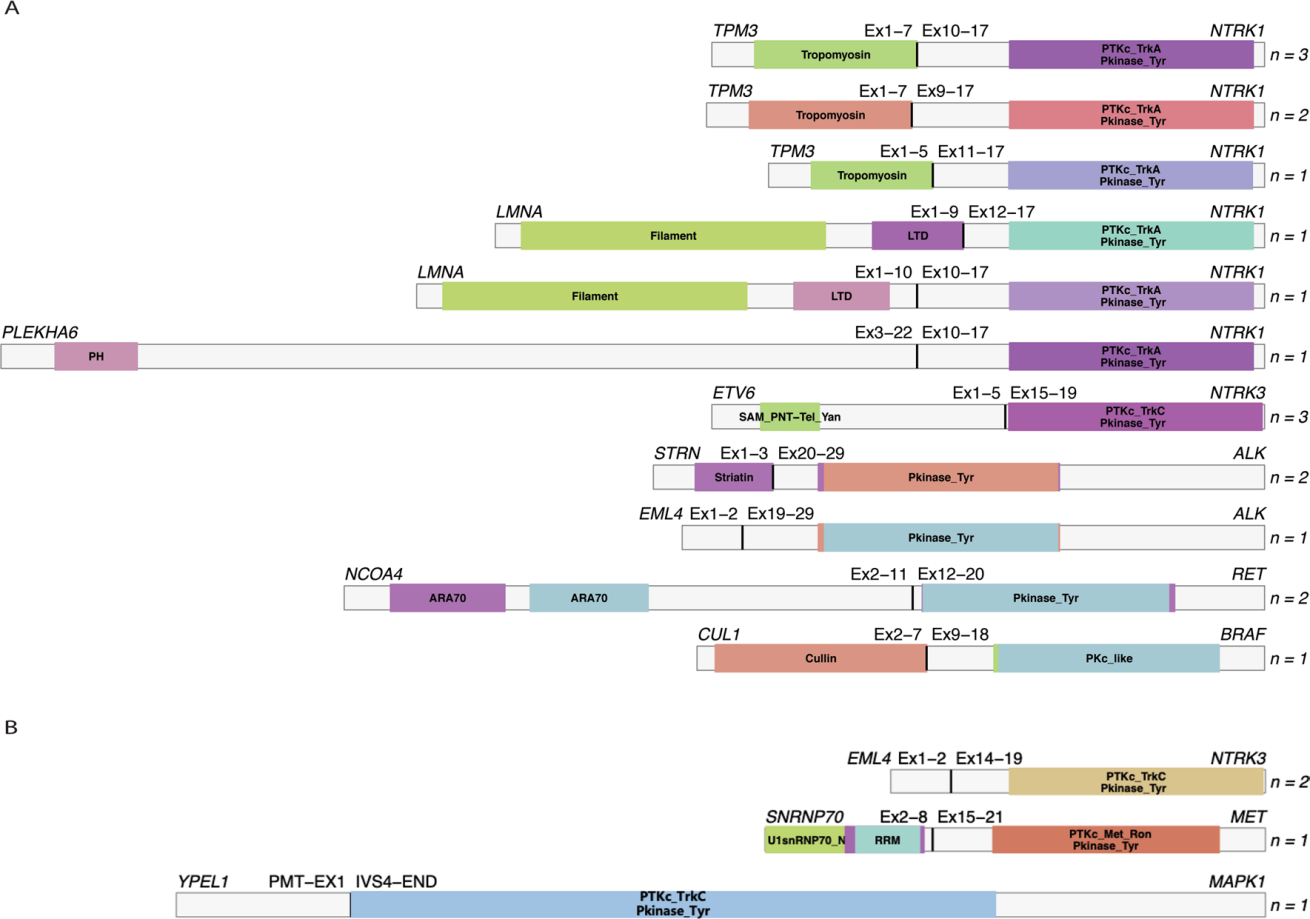
**A** Schematic representation of the predicted products of the 18 gene fusions detected by DNA NGS. **B** Schematic representation of the predicted products of four gene fusions detected by RNA NGS, but not by DNA NGS

DNA NGS was conducted in all 193 dMMR tumors, and identified eighteen genetic fusions (detailed in Additional file 2: Figure S1 and summarized in Fig. 1A). All gene fusions were exclusively presented in tumors harboring *MLH1* promoter hypermethylation and lacking concurrent *BRAF* or *RAS* driver mutations. These fusion-positive cases by DNA NGS represented 9% (18/193) of all dMMR tumors, 19% (18/91) of *MLH1*^me+^ tumors, and 46% (18/39) of *MLH1*^me+^ tumors with wild type *BRAF* or *RAS*. *NTRK1* fusions were the most frequent fusion events detected by DNA NGS, presenting in nine cases. All *NTRK1* fusions were intrachromosomal rearrangements involving known *NTRK1* partners. Six of these cases (6/9, 67%) harbored *TPM3-NTRK1* fusions with three different fusion breakpoints: exon(e)7 to e10 (3/9, 33%), e7 to e9 (2/9, 22%) and e5 to e11(1/9, 11%). *LMNA-NTRK1* fusions were found in two cases, with e9 to e12 and e10 to e10 fusion breakpoints, respectively. *PLEKHA6-NTRK1* fusion with e22 to e10 fusion breakpoint was found in one case. *NTRK3* gene fusions were identified in three cases, which were interchromosomal translocations with identical fusion breakpoints involving *ETV6* e1–5 on chromosome 12 and *NTRK3* e15–20 on chromosome 15. In-frame *ALK* gene rearrangements were found in three cases. Two of them were well-reported fusions connecting *STRN* e3 to *ALK* e20. Another one showed a fusion between *EML4* e1–2 and atypical breakpoint at *ALK* e19. *NCOA4-RET* fusion gene involving *NCOA4* e1–11 and *RET* e12–19 were observed in two cases. *CUL1-BRAF* fusion gene were found in one case, with the *BRAF* breakpoint located in intron 8, preserving the portion encoding the *BRAF* kinase domain.

Additional RNA NGS was performed in 21 *MLH1*^me+^ CRCs where neither oncogenic gene fusions nor *BRAF/RAS* driver mutations were detected by DNA NGS. Gene fusions were identified by RNA NGS in four (4/21, 19%) cases (detailed in Additional file 3: Figure S2 and summarized in Fig. 1B). Among them, two cases presented *EML4-NTRK3* fusions, which were formed through reciprocal translocation that joined the e1–2 of *EML4* with e14–19 of *NTRK3*. One case showed *MET* gene rearrangement involving a novel partner gene *SNRNP70*, with fusion breakpoints of *SNRNP70* e8 to *MET* e15. In another case, a novel in-frame fusion involving *YPEL1* and the extracellular signal-regulated kinase gene *MAPK1* was detected. This *YPEL1-MAPK1* chimeric transcript contained only part of the *MAPK1* C-terminal kinase domain by connecting e1 of *YPEL1* to e5 of *MAPK1*. *EML4-NTRK3* fusion was validated by RT-PCR and Sanger sequencing on FFPE samples of two cases. (Additional file 4: Figure S3).

**Fig. 2:**
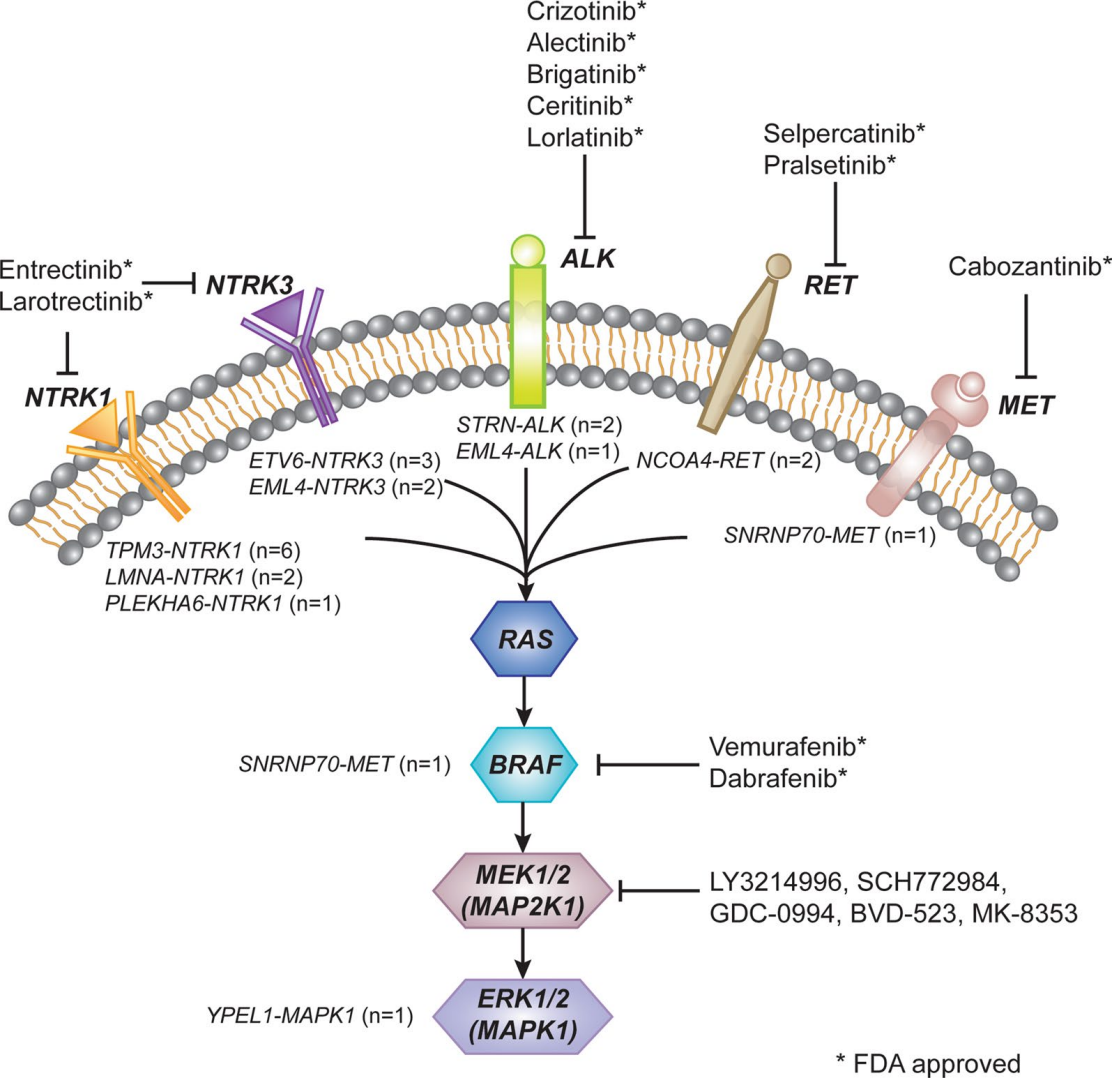
Schematic representation showing the activation of RTK-RAS signaling pathway by 22 gene fusions in our colorectal carcinoma cohort. All of the detected gene rearrangements within *NTRK1*, *NTRK3*, *ALK*, *RET*, *MET*, *BRAF* and *MAPK1* are targetable with currently available small molecule kinase inhibitors

All 22 fusion events were identified as driver alterations within RTK-RAS signaling pathway (Fig. 2). The majority of fusions affected upstream receptor tyrosine kinases (RTKs), including *NTRK1*(9/22, 41%), *NTRK3*(5/22, 23%), *ALK*(3/22, 14%), *RET*(2/22, 9%) and *MET*(1/22, 5%). Two other fusions involved components of mitogen-activated protein kinase (MAPK) cascade *BRAF*(1/22, 5%) and *MAPK1* (1/22, 5%), functioning in intracellular signal transduction of RTK-RAS pathway.

### Development of screening strategy for gene fusions in CRC using integrative DNA NGS and RNA NGS

**Fig. 3:**
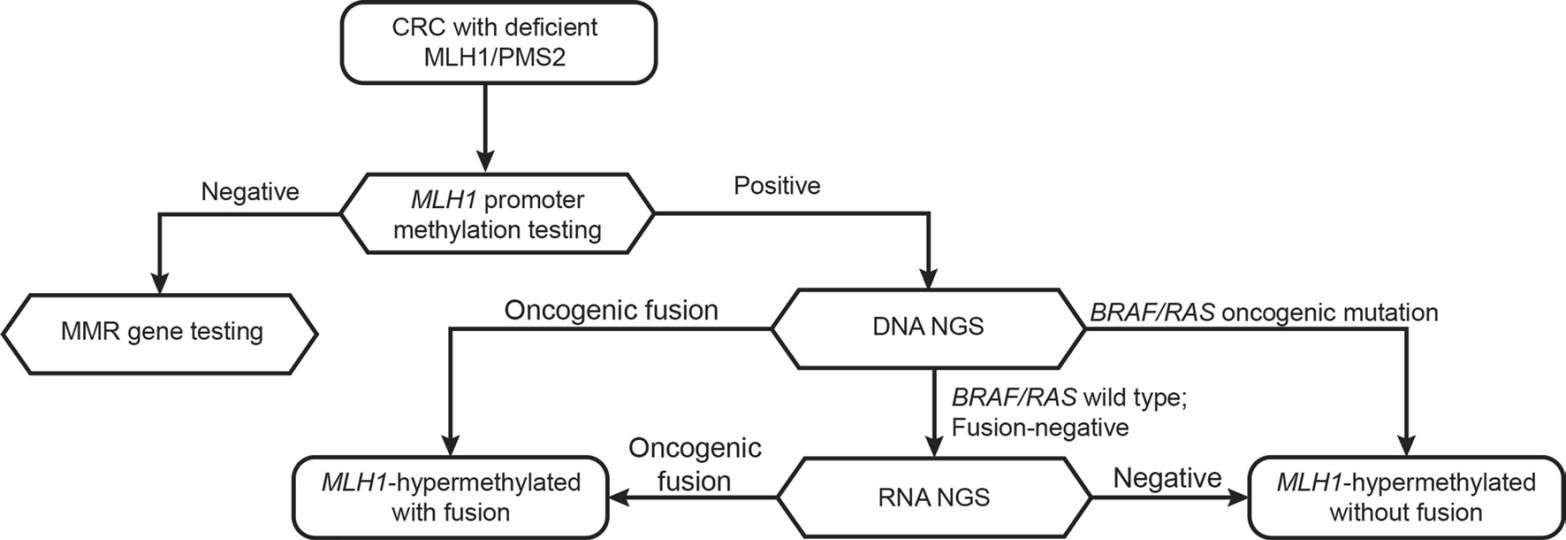
An optimized strategy incorporating RNA next generation sequencing to screen for gene fusions in colorectal carcinomas. CRC, colorecta carcinoma; NGS, next generation sequencing; MMR, mismatch repair

Comparing to DNA NGS alone, additional RNA NGS increased the proportion of detected fusion-positive tumors from 9% (18/193) to 11% (22/193) in dMMR cases, 19% (18/91) to 24% (22/91) in *MLH1*^me+^ dMMR cases, and from 46% (18/39) to 56% (22/39) in *MLH1*^me+^ *BRAF/RAS* wild-type dMMR cases, respectively. Based on these and our previously published findings, we developed an improved strategy with combined use of DNA NGS and RNA NGS to screen for potentially targetable gene fusions in CRCs (Fig. 3). In the molecular workup for *MLH1*^me+^ dMMR CRCs, when *BRAF/KRAS/NRAS* driver mutation testing was performed by DNA NGS, sequential RNA NGS was indicated when no gene fusions were found. Additionally, direct RNA NGS was suggested in *BRAF/RAS* wild-type cases when PCR assay was performed instead of DNA NGS for *BRAF/RAS* genotyping.

### Molecular genetic features of dMMR CRCs with gene fusions

**Fig. 4:**
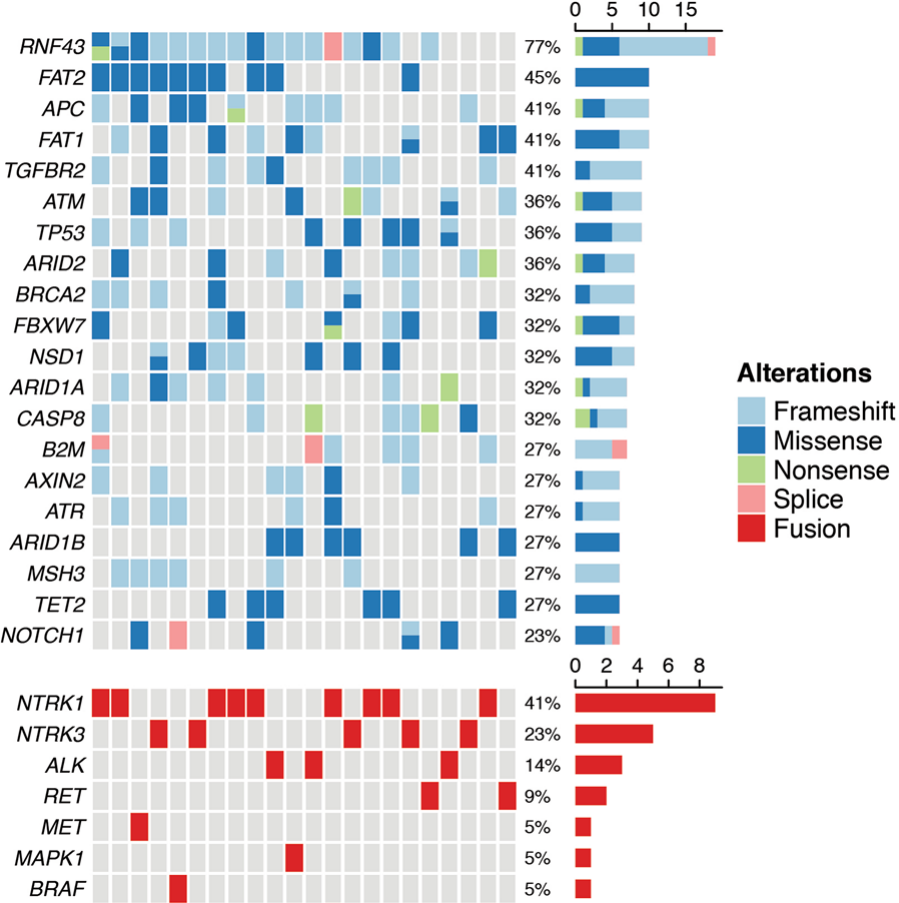
Mutation profile of top 20 most frequently mutated genes in 22 fusion-positive colorectal carcinomas. The significantly mutated genes are displayed as bar chart, ordered according to gene mutation frequencies (right plot). Different types of gene alterations in each tumor sample are displayed as heatmap (left plot)

*RNF43*(17/22, 77%), *FAT2*(10/22, 45%), *APC*(9/22, 41%), *FAT1*(9/22, 41%), *TGFBR2*(9/22, 41%), *ATM*(8/22, 36%), *TP53*(8/22, 36%), *ARID2*(8/22, 36%), *BRCA2*(7/22, 32%), *FBXW7*(7/22, 32%) and *ARID1A*(7/22, 32%) were identified as most recurrently mutated genes in dMMR CRCs harboring gene fusions (Fig. 4).

Alterations in key WNT pathway components were found in nineteen (19/22, 86%) cases. Apart from one *CTNNB1* activating mutation, these were primarily truncating mutations affecting various tumor suppressor genes *RNF43* (n = 17), *APC*(n = 9), *ARID1A*(n = 7), *FBXW7*(n = 7), *AXIN2*(n = 5), *TCF7L2*(n = 4), *FAM123B*(n = 3), and *SOX9* (n = 2). Nine (9/22, 41%) cases harbored frameshift mutations in *TGFBR2*, which encoded a key kinase receptor mediating TGF-β signaling transduction. However, few mutations affecting other key TGF-β pathway components *ACVR1B*, *SMAD2*, *SMAD3* and *SMAD4* were identified. In five (5/22, 23%) tumors, mutations in key genes of *PI3K* pathway were detected, including *PTEN* (n = 3), *PIK3CA* (n = 2), and *PIK3R1*(n = 1). Notably, both of the tumors with fusions affecting MAPK cascade components *BRAF* and *MAPK1* presented *PI3K* pathway aberrations (*PIK3CA* and *PTEN* mutation, respectively). None of the 22 tumors harbored mutations in other key *RTK-RAS* driver genes *BRAF*, *KRAS*, *NRAS*, *ERBB2* and *ERBB3*.

### Clinicopathological features of dMMR CRCs with gene fusions

**Fig. 5:**
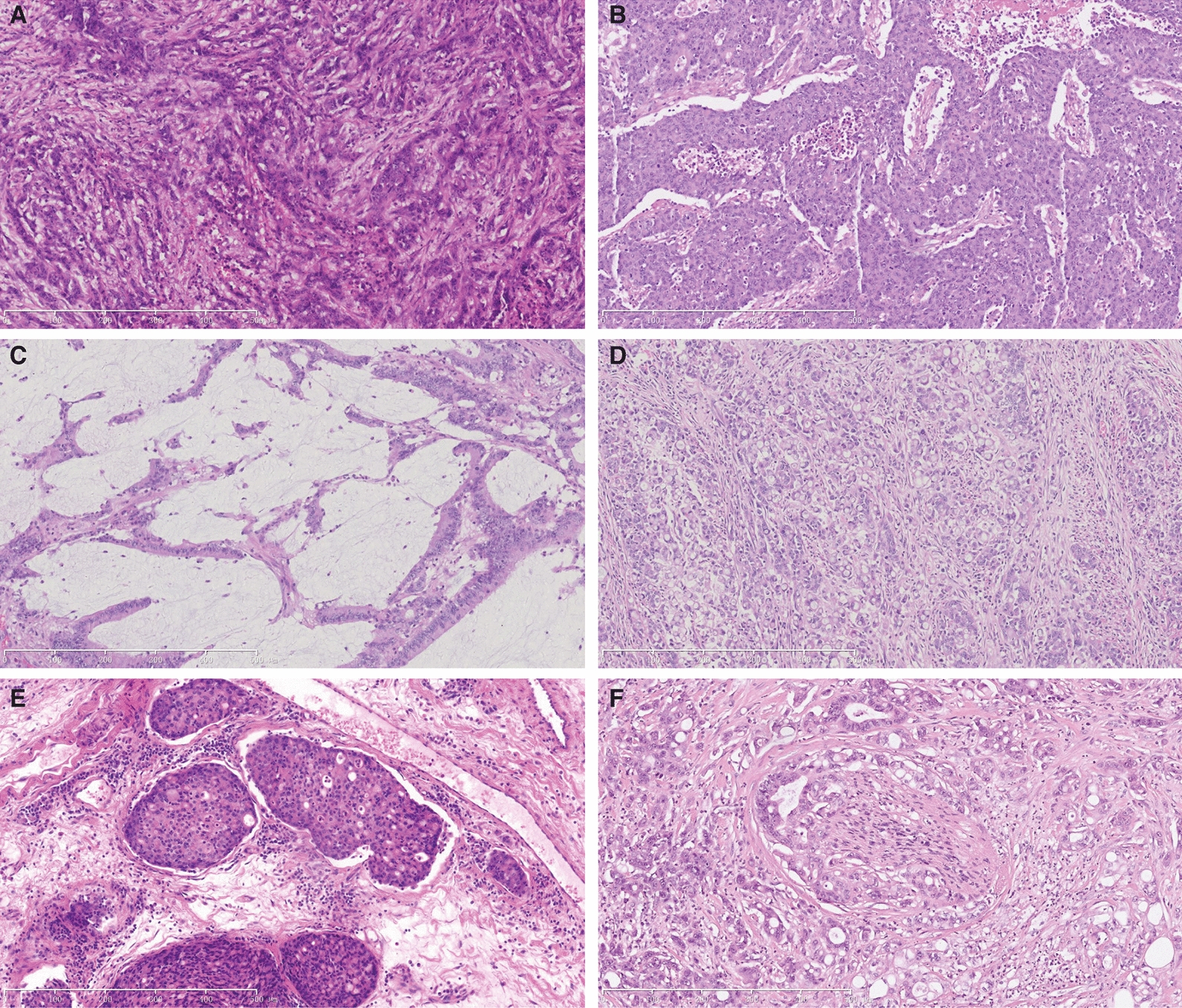
Histologic features of colorectal carcinomas harboring gene fusions. **A** Poorly differentiated area in tumor harboring *TPM3*(e7)–*NTRK1*(e10) fusion, showing ribbon-like growth pattern. **B** Poorly differentiated area in tumor harboring *TPM3*(e7)–*NTRK1*(e9) fusion, displaying vague nested growth pattern. **C** Mucinous differentiated area in a tumor harboring *ETV6*(e5)–*NTRK3*(e15) fusion. **D** Diffuse signet-ring mucinous component in a *LMNA*(e9)–*NTRK1*(e12) fusion tumor. **E** Lymphovascular invasion in a *TPM3*(e5)–*NTRK1*(e11) fusion tumor; **F** Perineural invasion in a *ETV6*(e5)–*NTRK3*(e15) fusion tumor

The clinicopathological features of 22 tumors harboring gene fusions detected by either DNA NGS or RNA NGS were listed in Table 1. The majority of these tumors were diagnosed in female (13/22, 59%). All patients were elderly over 50 years old, with the median age of 72 years. Tumors were predominantly right-sided (20/22, 91%), and over half were located at hepatic flexure (13/22, 59%). All tumors were either stage II (15/22, 68%) or stage III (7/22, 32%) according to TNM classification. Histologically, poorly differentiated areas were detected in more than half of these tumors (13/22, 59%). Nine cases (9/22, 41%) presented focal to extensive mucinous components, including one case displaying a diffuse signet-ring mucinous component. Lymphovascular invasion was observed in ten cases (10/22, 45%), and perineural invasion was observed in two cases (2/22, 9%) (Fig. 5). Within 91 *MLH1*^me+^ CRCs cases, patients with fusion-positive tumors were significantly older (median 72 vs. 62 years, *P* = 0.013) comparing with those harboring fusion-negative tumors. They also showed a significantly higher preponderance of hepatic flexure localization (59% vs. 12%, *P* < 0.001) and poor differentiation (55% vs. 23%, *P* = 0.019) (Table 2). No statistically significant differences in other clinicopathological features, including gender, stage, mucinous differentiation, lymphovascular and perineural invasion were observed between two groups.

**Table 1:** Clinicopathological features of 22 tumors harboring gene fusions detected by either DNA or RNA next generation sequencing

Case No.	Fusion type	Clinical feature					Histological feature			
		Age	Gender	Tumor location	TNM	AJCC stage	Tumor differentiation	Mucinous differentiation	Lymphovascular invasion	Perineural invasion
1	*TPM3*(e7)–*NTRK1*(e10)	61	Female	Ileocecum	T2N1bM0	II	Moderate–poor	Yes	No	No
2	*TPM3*(e7)–*NTRK1*(e10)	83	Female	Hepatic flexure	T3N1bM0	III	Moderate–poor	No	Yes	No
3	*TPM3*(e7)–*NTRK1*(e10)	77	Male	Hepatic flexure	T3N0M0	II	Moderate–low	Yes	No	No
4	*TPM3*(e7)–*NTRK1*(e9)	69	Female	Hepatic flexure	T3N0M0	II	Low	No	Yes	No
5	*TPM3*(e5)–*NTRK1*(e11)	76	Female	Hepatic flexure	T4aN2aM0	III	Low	No	Yes	Yes
6	*TPM3*(e7)–*NTRK1*(e9)	83	Female	Ascending colon	T2N0M0	II	Low	No	No	Yes
7	*LMNA*(e9)–NTRK1(e12)	75	Female	Ascending colon	T3N2bM0	III	Moderate–low	No	Yes	No
8	*LMNA*(e10)–*NTRK1*(e10)	82	Female	Ascending colon	T3N1cM0	III	Moderate–low	Yes	Yes	No
9	*PLEKHA6*(e22)–*NTRK1*(e10)	75	Female	Transverse colon	T3N1cM0	III	Moderate	No	Yes	No
10	*ETV6*(e5)–*NTRK3*(e15)	55	Male	Descending colon	T3N0M0	II	High	Yes	No	No
11	*ETV6*(e5)–*NTRK3*(e15)	59	Male	Hepatic flexure	T3N0M0	II	High–moderate	No	Yes	No
12	*ETV6*(e5)–*NTRK3*(e15)	53	Male	Hepatic flexure	T4aN0M0	II	Moderate–low	No	Yes	Yes
13	*EML4*(e2)–*NTRK3*(e14)	75	Male	Hepatic flexure	T3N0M0	II	Moderate	Yes	No	No
14	*EML4*(e2)–*NTRK3*(e14)	77	Female	Ascending colon	T3N0M0	II	Moderate	Yes	No	No
15	*STRN*(e3)–*ALK*(e20)	69	Female	Hepatic flexure	T3N0M0	II	Moderate–poor	No	Yes	No
16	*STRN*(e3)–*ALK*(e20)	69	Female	Ascending colon	T2N0M0	II	Moderate	Yes	No	No
17	*EML4*(e2)–*ALK*(e19)	62	Female	Hepatic flexure	T3N1bM0	III	Moderate–poor	No	Yes	No
18	*NCOA4*(e11)–*RET*(e12)	71	Female	Hepatic flexure	T3N0M0	II	Moderate	Yes	No	No
19	*NCOA4*(e11)–*RET*(e12)	82	Male	Hepatic flexure	T3N0M0	II	Moderate–low	No	No	No
20	*SNRNP70*(e8)–*MET*(e15)	82	Male	Hepatic flexure	T3N0M0	II	Moderate–low	Yes	No	No
21	*CUL1*(e7)–*BRAF*(e9)	75	Male	Splenic flexure	T3N0M0	II	Moderate	No	No	No
22	*YPEL1*(e1)–*MAPK1* (e5)	79	Male	Hepatic flexure	T3N0M0	II	Moderate	No	No	No

**Table 2:** Comparison of clinicopathological features between fusion-positive MLH1 hypermethylated colorectal cancers, and fusion-negative MLH1 hypermethylated colorectal cancers

	*MLH1*^me+^ with fusion (n = 22)	*MLH1*^me+^ without fusion (n = 69)	*P*^*#*^
Age, median, years	72	62	0.013
Gender, n(%)			
Female	13 (59)	37 (54)	0.806
Male	9 (41)	32 (46)	
Tumor location, n(%)			
Hepatic flexure	13 (59)	8 (12)	< 0.001
Other sites of large intestine	9 (41)	61 (78)	
Ileocecum	1 (5)	20 (29)	
Ascending colon	5 (23)	16 (23)	
Transverse colon	1 (5)	6 (9)	
Splenic flexure	1 (5)	2 (3)	
Descending colon	1 (5)	5 (7)	
Rectosigmoid	0	12 (17)	
AJCC stage, n(%)			
I	0 (0)	4 (5)	0.925
II	15 (68)	42 (61)	
III	7 (32)	20 (29)	
IV	0 (0)	2 (3)	
Presence of poor differentiated area, n(%)			
Yes	13 (59)	18 (26)	0.009
No	9 (41)	51 (74)	
Mucinous differentiation, n(%)			
Yes	9 (41)	25 (36)	0.801
No	13 (59)	44 (64)	
Lymphovascular invasion, n(%)			
Yes	10 (45)	23 (33)	0.445
No	12 (55)	46 (67)	
Perineural invasion, n(%)			
Yes	3 (14)	5 (7)	
No	19 (86)	64 (93)	

*MLH1*^me+^, *MLH1* hypermethylated

^#^*P* values were based on Chi-square test, Fisher’s exact test, or Mann–Whitney tests, whenever appropriate. All statistical tests were two-sided

## Discussion

It has been documented in our previous study that oncogenic fusions were significantly enriched in dMMR CRCs harboring hypermethylated *MLH1* and wild-type *BRAF/RAS* [10]. Herein, we conducted further study using integrative DNA and RNA sequencing, aimed for more accurate and comprehensive characterization of gene fusions in CRCs. We proved that RNA NGS was a valuable addition to DNA NGS for enhancing fusion detection (46–56% in *MLH1*^me+^ *BRAF/RAS* wild-type dMMR CRCs), as well as identifying novel or atypical fusion types. An optimizing strategy incorporating RNA NGS to screen for oncogenic fusions in CRCs was thus proposed. Next, we presented a detailed analysis of molecular genetic profile and clinicopathological features of fusion-positive dMMR CRCs. All fusions involved RTK-RAS signaling pathway, predominantly RTKs, and were mutually exclusive to other RTK-RAS driver mutations. WNT pathway alterations were also frequently detected. Fusion-positive tumors were typically diagnosed in elder patients, predominantly right-sided, preferentially occurred at hepatic-flexure and showed histologically poor-differentiated components.

Considering the distinct advantages over other techniques in gene fusion detection, the latest National Comprehensive Cancer Network guideline for non-small cell lung cancer recommended RNA-based NGS in patients with no identifiable driver oncogenes detected by broad panel DNA NGS [19]. In the present study, we revealed that nearly 20% (n = 4) *MLH1*^me+^ dMMR tumors with neither oncogenic fusions nor *BRAF/RAS* driver mutations detected by DNA NGS were positive for gene fusions by RNA NGS. In all of these four cases, the genomic breakpoints were located at large introns or intronic repetitive elements, which were typically not sufficiently covered by large hybrid-capture based DNA NGS panel. In our cohort, fusion-positive tumors by integrative DNA and RNA NGS represented 11% of dMMR cases, 24% of *MLH1*^me+^ dMMR cases, and 56% of *MLH1*^me+^ dMMR cases with wild-type *BRAF/RAS*. These proportions were much higher in comparison to that reported in prior DNA-based large-scale clinical research using MSK-IMPACT assay [9], suggesting that optimizing fusion detection process by incorporating additional RNA NGS was able to achieve a considerably higher yield of gene fusions in CRCs. In addition, RNA NGS successfully identified two potentially actionable kinase fusions (*SNRNP70-MET* and *YPEL1-MAPK1*) which have not been reported in CRCs before. Therefore, we suggested the sequentially combined use of DNA NGS and RNA NGS as a highly effective strategy to uncover oncogenic gene fusions in *MLH1*^me+^ CRCs, which were suggested as markers for unfavorable prognosis and targets for personalized therapy [20]. In clinical settings where *BRAF/RAS* PCR was applied as an alternative to DNA NGS, direct RNA NGS was recommended in *BRAF/RAS* wild-type cases for maximized cost-efficiency.

RNA extracted from fresh-frozen (FF) tissue was preferentially used for gene expression study. However, the availability of FF tissue was very limited in clinical practice. FFPE specimens represent more accessible and exploitable sources for molecular studies. Despite that RNA isolated from FFPE samples often suffer degradation and chemical modification due to fixation and archiving method, recent comparative studies have reported high correlation of RNA NGS detected gene expression profile between paired FFPE and FF samples [21, 22]. Notably, artifacts introduced during library preparation and sequence alignment might hamper the reliable prediction of gene fusions by RNA NGS, leading to unaligned or out-of-frame transcripts. In clinical practice, sequential cross-validation using PCR or Sanger sequencing might be considered for RNA-NGS detected novel fusions, especially those with low abundance transcripts and with multiple breakpoints within the same exon of the fusion partner [22].

Aberrant activation of *RTK-RAS* signaling pathway has been well-recognized as key molecular event in CRC tumorigenesis. Previously, among *MLH1*^me+^ dMMR CRCs, *RTK-RAS* activation was generally considered to be mediated by *BRAF* oncogenic mutation, occurring at the early stage of serrated neoplasia pathway [23]. In this and our prior studies [14], we revealed that almost all gene fusions were detected in dMMR CRCs harboring hypermethylated *MLH1*, which presented as the only RTK-RAS driver alteration in these tumors. It is rational to suggest gene fusions as one major mechanism of RTK-RAS oncogenic activation in *MLH1*^me+^ dMMR CRCs, second only to *BRAF* mutation. Most of the fusion-positive cases harbored RTK fusions susceptible to tyrosine kinase inhibition therapy. In spite of the rarity, it is worth noting that a minority of fusions involved *MAP3K(BRAF)* and *MAP1K*, genes encoding key components of downstream mitogen-activated protein kinase (MAPK) cascade which were essential for intracellular RTK-RAS signal transduction. Due to the potential feedback activation of *EGFR* [24, 25], combination therapy consisting of both *EGFR* and *RAS/RAF* inhibitors might be required in these cases [26–28].

Despite that dMMR was typically considered as a favorable prognostic marker in CRC patients, oncogenic fusions have been shown to be associated with poorer clinical outcome [29, 30]. The detected genetic fusions primarily affected RTKs, and rendered those tumors amenable to FDA approved targeted therapy that might reverse the otherwise poor prognosis. Therefore, efficient identification and detailed characterization of fusion variants is of key clinical significance. In our dMMR CRC cohort, TRK fusions, particularly *NTRK1* fusions, were the most frequently detected fusion events. We observed that *TPM3* was the most common fusion partner of *NTRK1* in CRCs (66%), which was in consistent with previous reports [31, 32]. *NTRK1-LMNA* and *NTRK1-PLEKHA6*, two other *NTRK1* fusion types documented in CRCs before [31], were found to take a lesser proportion in our cases. We did not detect *NTRK1* fusions with *SCYL3* and *TPR*, which have been reported rarely before [32]. In previously published reports, *NTRK3* fusions were found in only a few CRCs, accounting for two out of 21 fusion events in cases assessed by MSK-IMPACT testing [9], and one out of 16 *NTRK* fusion events in cases screened by pan-TRK IHC testing [32]. However, it has been implicated that substantial numbers of *NTRK3* gene rearrangements occurred at large introns (*NTRK3* intron 13 and 14), and might be omitted by DNA NGS alone [7]. Also, large scale clinical researches have documented a lower sensitivity of pan-TRK IHC assay for *NTRK3* fusions comparing to *NTRK1/2* fusions [33, 34]. In the present study, using sequentially combined DNA NGS and RNA NGS, we observed a much higher proportion of *NTRK3* fusions in all detected fusion events (5/22). This finding further justified incorporating RNA NGS in clinical practice to more efficiently identify fusion-positive tumors, especially those harboring *NTRK3* fusions. Although several rare *NTRK3* fusion types were previously identified in CRCs, including *KANK1-NTRK3*, *COX5A-NTRK3* and *VPS18-NTRK3* [11, 32], here we observed that *NTRK3* exclusively formed fusion with its main partner gene *ETV6* or *EML4*. As far as we can see, two of the gene fusions affecting RTKs presented in our cohort were not well-documented in CRCs previously. An *EML4-ALK* fusion was found to involve atypical *ALK* breakpoint within exon 19 that encoded transmembrane domain. *ALK* rearrangements at exon 19, instead of usual site within intron 19 or exon 20, has only been rarely described in malignant stromal sarcoma [35] and lung adenocarcinoma [36, 37] before. Except for a case demonstrating a partial response to targeted therapy [36], reports on clinical implication of this breakpoint were very limited. A *MET* fusion with novel partner gene *SNRNP70* encoding a key component of spliceosome was identified in one case. Although *MET* gene copy number gain and protein overexpression were proved to drive CRC tumor malignant progression [38], *MET* gene fusions have not been noted in CRCs before.

Apart from RTKs, gene fusions involving the downstream MAPK cascade were also potentially actionable. Both of the two fusions affecting MAPK cascade detected in our cohort have been rarely reported before. The *CUL1*(e7)–*BRAF*(e9) fusion was previously observed in a few cases of melanoma [39] and low-grade serous carcinoma (LGSC) [40], and only once in CRC [9]. Tumor cells harboring *CUL1-BRAF* fusion have been found to show activation of MAPK signaling pathway and sensitivity to *MEK/RAF* inhibition. Moreover, complete response to *MEK* inhibitor-based combination therapy was noted in one LGSC patient bearing *CUL1-BRAF* fusion [40]. The *YPEL1*(e1)–*MAPK1* (e5) was a novel fusion to our limited knowledge. Typically, abnormal overactivation of *MAPK1 (ERK)* was induced by hyperactivated upstream *RTK/RAS* signaling. Gain-of-function mutations in the gene itself were only seldomly documented in laboratory models or in clinical cases [41]. Since only part of *MAPK1* C-terminal kinase domain was involved in the detected *YPEL1-MAPK1* chimeric transcript, whether this fusion gene possessed oncogenic properties awaited further investigation. Given that constitutively activated RTK fusions could concurrently induce downstream RAS and PI3K pathways, it is not surprising to find the general low frequency of *PI3K* pathway aberration among tumors harboring RTK fusions. However, *PIK3CA* and *PTEN* mutations were observed in these two cases with fusions involving MAPK cascade. This finding indicated that despite the well-established intimate intersection of RTK downstream pathways RAS-MAPK and PI3K-mTOR, constitutive activation of MAPK cascade by gene rearrangements might not be sufficient to cross-activate PI3K-mTOR signaling and give rise to malignant transformation events.

We observed that *RNF43* was the most frequently mutated one among all genes analyzed in this study. This result strengthened our previous finding that *RNF43* inactivation was directly correlated with *MLH1* hypermethylation, instead of *BRAF* mutation status [14]. Nearly 90% of the fusion-positive cases were presented with WNT pathway alterations. Additionally, four out of 12 top recurrently mutated genes (*RNF43*, *APC*, *FBXW7* and *ARID1A*) were found to be involved in WNT signaling. It is rational to assume that synergistic cooperation of WNT pathway components might play an important role in tumorigenesis of fusion-positive CRCs. A very recent in vitro study revealed susceptibility to poly (ADP-ribose) polymerase (PARP) inhibitors in a subset of poor prognostic CRCs with DNA homologous recombination repair (HRR) pathway deficiency [42]. Our data showed that one third of fusion-positive tumors harbored mutations in crucial HRR genes *ATM* and *BRCA2*, and lay a rationale for further clinical studies investigating PARP inhibitors as a potential therapeutic option for these tumors.

Based on large sample size and detailed molecular subclassification, we further conducted comparison between fusion-positive and fusion-negative tumors within *MLH1*^me+^ CRCs. Fusion-positive tumors were found to exhibit characteristic clinicopathological features, including old age, preferential hepatic flexure localization and poor differentiation. Typically, dMMR tumors were considered as a relatively homogeneous molecular entity characterized by vulnerability to immunotherapy, which have recently been approved by FDA as first-line treatment for metastatic dMMR CRCs. Our findings highlighted the delicate yet noticeable heterogeneity within dMMR CRCs, and justified more precise molecular subtyping for personalized diagnosis and therapy in CRCs. In addition, a recent study has uncovered the continuum variation of tumor molecular profile along the large intestine, and necessitated more precise classification of CRCs by tumor location [43]. In this study, we not only confirmed that fusion-positive CRCs were primarily right-sided lesions, but also specified that more than half of them were localized at hepatic flexure. In clinical practice, these results implicated that CRC patients with above-mentioned clinicopathological features might be prioritized for molecular assay for gene fusions, including RNA NGS.

In the present study, we found that fusion-positive tumors showed a significantly higher preponderance of hepatic flexure localization. Variations of microbiome, clinicopathological features and molecular profiles have been reported to be associated with primary tumor localization along the large intestine. Several studies have documented the emerging role of gut microbiota in CRC formation and progression [43, 44]. However, as far as we know, the microbiome characterization of hepatic flexure has not been well described. The mechanism underlying the preferential localization of fusion-positive in hepatic flexure remained to be further explored.

In summary, our study presented a practical and highly effective screening procedure for genetic fusions through integrated DNA NGS and RNA NGS in a selected subset of dMMR CRCs harboring hypermethylated *MLH1*. With a detailed description of fusion variants, molecular profile and clinicopathologic features, we further characterized fusion-positive CRCs as a distinctive subtype with key clinical significance.

## Supplementary Information

**Additional file 1: Table S1.** List of genes included in the 1021 genes panel.

**Additional file 2: Figure S1.** Schematic representation of the predicted products of the 18 gene fusions detected by DNA NGS.

**Additional file 3: Figure S2.** Schematic representation of the predicted products of the four gene fusions detected by RNA NGS.

**Additional file 4: Figure S3.** Validation of EML4-NTRK3 fusion in sample 0394 and sample 0447 using RT-PCR (top panel) and Sanger sequencing (bottom panel). The sequence spanning the break point is 5′- CAGTCTCAAGTAAAG- GTCCCGTGGCTGTCA-3′, which confirms the fusion identified by our assay.

## Data Availability

The datasets used, generated, and analyzed during the present study are available from the corresponding authors on reasonable request.

## Abbreviations

CRC: Colorectal carcinoma
FISH: Fluorescence in situ hybridization
RT-PCR: Real-time polymerase chain reaction
NGS: Next generation sequencing
dMMR: Mismatch repair deficient
*MLH1*^me^^+^: Hypermethylated *MLH1*
FFPE: Formalin-fixed paraffin-embedded
rRNA: Ribosomal RNA
e: Exon
RTKs: Receptor tyrosine kinases
MAPK: Mitogen-activated protein kinase
LGSC: Low-grade serous carcinoma
PARP: Poly ADP-ribose polymerase
