# Supplementary figures and images for: Comprehensive analysis of oncogenic fusions in mismatch repair deficient colorectal carcinomas by sequential DNA and RNA next generation sequencing
Source: J Transl Med. 2021 Oct 17;19:433. doi: 10.1186/s12967-021-03108-6 (PMC8522100; doi:10.1186/s12967-021-03108-6)

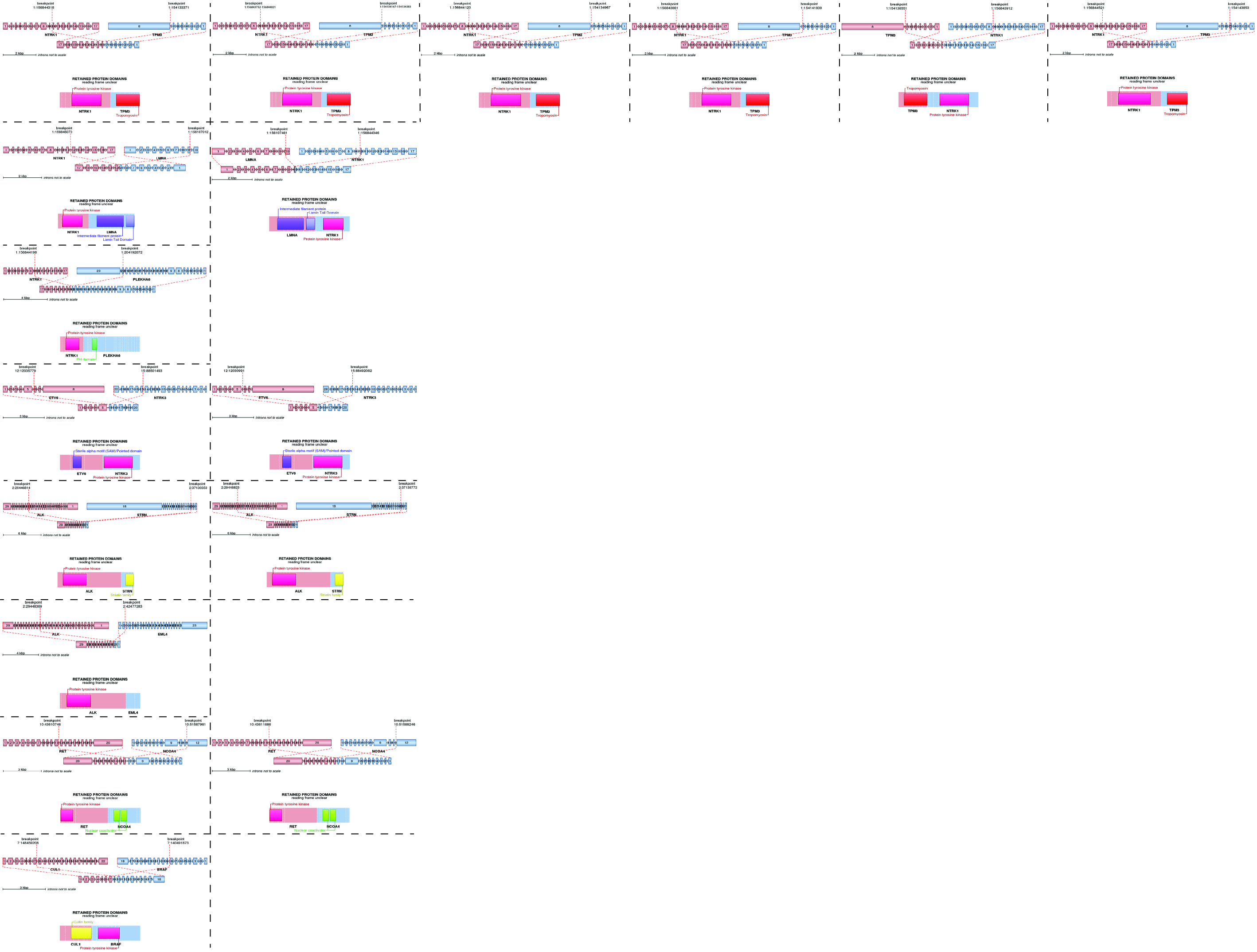

Supplement: Supplementary file 2 — Additional file 2: Figure S1. Schematic representation of the predicted products of the 18 gene fusions detected by DNA NGS. [file 12967_2021_3108_MOESM2_ESM.tif]

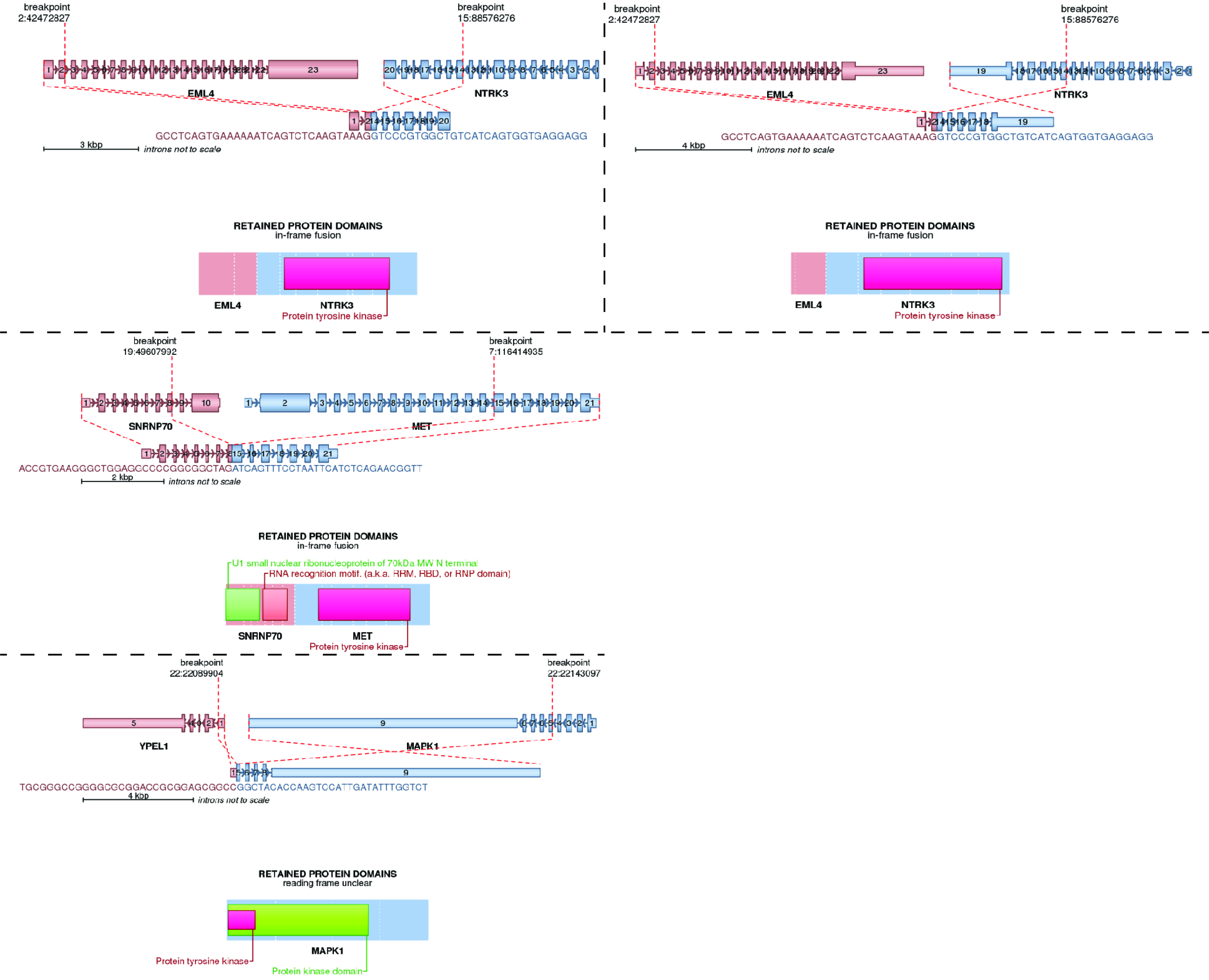

Supplement: Supplementary file 3 — Additional file 3: Figure S2. Schematic representation of the predicted products of the four gene fusions detected by RNA NGS. [file 12967_2021_3108_MOESM3_ESM.tif]

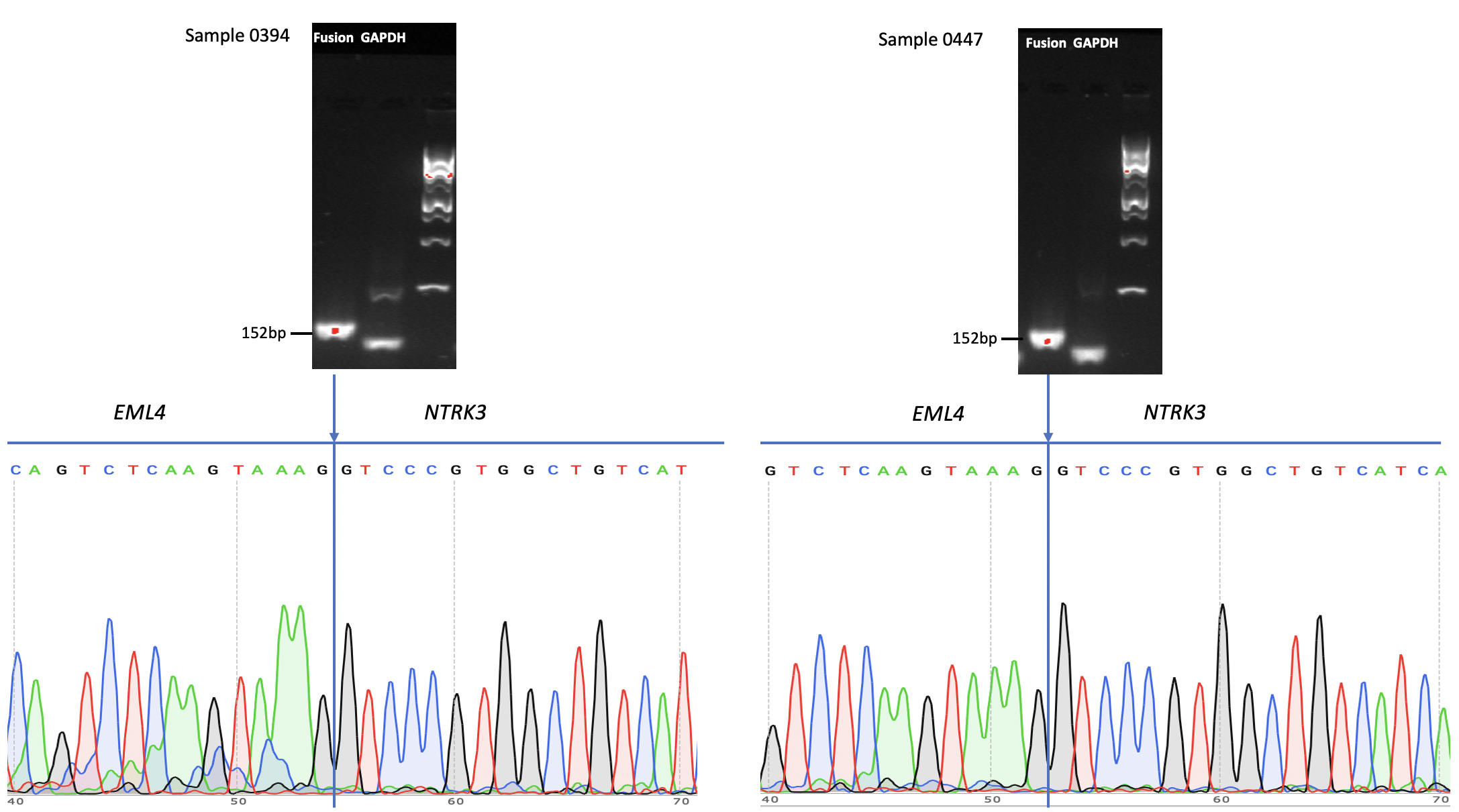

Supplement: Supplementary file 4 — Additional file 4: Figure S3. Validation of EML4-NTRK3 fusion in sample 0394 and sample 0447 using RT-PCR (top panel) and Sanger sequencing (bottom panel). The sequence spanning the break point is 5′- CAGTCTCAAGTAAAG- GTCCCGTGGCTGTCA-3′, which confirms the fusion identified by our assay. [file 12967_2021_3108_MOESM4_ESM.png]
